# A Novel* CRYBB2* Stopgain Mutation Causing Congenital Autosomal Dominant Cataract in a Chinese Family

**DOI:** 10.1155/2016/4353957

**Published:** 2016-11-29

**Authors:** Yu Zhou, Yaru Zhai, Lulin Huang, Bo Gong, Jie Li, Fang Hao, Zhengzheng Wu, Yi Shi, Yin Yang

**Affiliations:** ^1^Key Laboratory for Human Disease Gene Study, Sichuan Academy of Medical Sciences and Sichuan Provincial People's Hospital, School of Medicine, University of Electronic Science and Technology of China, Chengdu 610072, China; ^2^Center of Information in Biomedicine, University of Electronic Science and Technology of China, Chengdu 610072, China; ^3^Chinese Academy of Sciences Sichuan Translational Medicine Research Hospital, Chengdu 610072, China; ^4^Department of Ophthalmology, Sichuan Academy of Medical Sciences and Sichuan Provincial People's Hospital, Chengdu 610072, China

## Abstract

Congenital cataract is the most common cause of the visual disability and blindness in childhood. This study aimed to identify gene mutations responsible for autosomal dominant congenital cataract (ADCC) in a Chinese family using next-generation sequencing technology. This family included eight unaffected and five affected individuals. After complete ophthalmic examinations, the blood samples of the proband and two available family members were collected. Then the whole exome sequencing was performed on the proband and Sanger sequencing was applied to validate the causal mutation in the two family members and control samples. After the whole exome sequencing data were filtered through a series of existing variation databases, a heterozygous mutation c.499T<G (p.E167X) in* CRYBB2 *gene was found. And the results showed that the mutation cosegregated with the disease phenotype in the family and was absolutely absent in 1000 ethnicity-matched control samples. Thus, the heterozygous mutation c.499T<G (p.E167X) in* CRYBB2* was the causal mutation responsible for this ADCC family. In conclusion, our findings revealed a novel stopgain mutation c.499T<G (p.E167X) in the exon 6 of* CRYBB2* which expanded the mutation spectrum of* CRYBB2* in Chinese congenital cataract population and illustrated the important role of* CRYBB2* in the genetics research of congenital cataract.

## 1. Introduction

Cataract, which often leads to visual impairment or blindness, can be unilateral or bilateral, congenital, or acquired [1]. Congenital cataract is one of the most common visual disorders during infancy or childhood and approximately affected 1–6 and 5–15 per 10,000 birth in industrialized and poor areas, respectively [2–5]. Any factors affecting the development of the fetalis lens might lead to occurrence of congenital cataract, such as heredity, the matrix virus infection in pregnancy, and pregnant women suffering from metabolic diseases. Congenital cataract is often characterized by clinical and genetic heterogeneity [5]. Different mutations can cause similar cataract phenotypes, such as the Coppock-like cataract mutations [6, 7], while the same variation may result in different cataract patterns such as the mutation p.Q155X in* CRYBB2* gene [8, 9]. Multiple genetic studies revealed that about one-third of congenital cataract was associated with genetic factor [10]. Up to now, an increasing number of loci and genes (over 44 loci and 38 genes, resp.) were identified to be associated with congenital cataract [11–14]. Among these mutations, about half are in crystalline genes, mainly including* CRYAA*,* CRYAB*,* CRYBA4*,* CRYBB1*,* CRYBB2*, and* CRYGC* [15]; one-quarter of mutations are in connexin genes, such as* GJA3* and* GJA *[16, 17]; the remaining mutations are other genes which consist essentially of* HSF4* [18],* MIP *[19], and* EPHA2* [20]. Although autosomal dominant congenital cataract (ADCC) is the major inherited mode of congenital cataract, there were a few reports about autosomal recessive [21–23] and X-linked inheritance mode [24, 25].

Our study is a genetic study in which we attempted to identify the disease gene in a four- generation Chinese family with autosomal dominant congenital cataract through the exome sequencing and direct sequencing. After analysis and validation, a novel mutation c.499T<G, in the exon 6 of* CRYBB2*, resulting in a truncation of 39 amino acids from the COOH-terminal end of *β*B2-crystallin, was identified to be the disease causing mutation for the congenital cataract in this family. These findings provided evidence to expand the mutation spectrum of* CRYBB2* in the Chinese congenital cataract population and we could further offer some clues to the molecular genetic mechanism of congenital cataract.

## 2. Materials and Methods

### 2.1. Subjects and Clinical Evaluation

This study was approved by the Institutional Review Board of the Hospital of University of Electronic Science and Technology of China and Sichuan Provincial People's Hospital. All the participants were Han Chinese. Written informed consent was obtained in adherence to the Declaration of Helsinki for all the subjects or their guardians prior to the study. A four-generation Chinese family with congenital cataract from Sichuan Province of China consisting of thirteen members (eight unaffected and five affected individuals) and 1000 ethnicity-matched control subjects were recruited from the Hospital of University of Electronic Science and Technology of China and Sichuan Provincial People's Hospital. Complete ophthalmic examinations were applied in the three members of the family (III:5, III:6, and IV:1) including visual acuity (by standard logarithmic visual acurity chart), intraocular pressure (by noncontact ophthalmotonometer), slit-lamp ophthalmoscopy, and indirect ophthalmoscopy under dilated pupils (by the supplementary lens). The ethnicity-matched control subjects were with no family history or eye diseases. This is a genetic study in which we used the genetic method to illustrate the cause of congenital cataract (the heritage disease). Clinical information about the three members in the family was listed in the Table 1.

### 2.2. DNA Samples

Venous blood of each participant including III:5, III:6, and IV:1 was obtained from cubital vein and collected in an EDTA tube. The total genomic DNA was extracted by using a blood DNA extraction kit according to the protocol provided by the manufacturer (TianGen, Beijing, China). DNA integrity was evaluated by 1% agarose gel electrophoresis. Then all DNA samples were stored at −20°C until being used.

### 2.3. Exome Screening of the Proband

Whole exome sequencing technology is a useful tool for identifying disease causing mutations which could target the coding regions of the human DNA. In this study, the genomic DNA sample of the proband in this family was subjected to the exome sequencing by Axeq Technology Inc., Seoul, Korea. The sample was prepared strictly in accordance with the Illumina protocols of Sure Select Target Enrichment System Capture Process. Briefly, the genomic DNA sample was randomly fragmented according to the principle of nebulization. Then, the 250–300 bp fragments of DNA were subjected to three enzymatic steps: end repair, A-tailing, and adapter ligation. Afterwards, the specific product was amplified by ligation-mediated polymerase chain reaction (PCR) and validated using the Agilent Bioanalyzer. Each captured library was then loaded onto the Illumina HiSeq2000 sequencer. Then we use Illumina base calling software V1.7 to analyze the raw image files with default parameters.

### 2.4. Read Mapping and Variant Analysis

Briefly, the read was mapped against UCSC hg19 (http://genome.ucsc.edu/) by BWA (http://bio-bwa.sourceforge.net/). The SNPs and Indels are detected by SAMTOOLS (http://samtools.sourceforge.net/). Single-nucleotide polymorphism (SNP) analysis was performed as follows. (1) Reads was aligned to the NCBI human reference genome (gh19/NCBI 37.1) with SOAPaligner method V2.21. (2) For paired-end read with duplicated start and end sites, only one copy with the highest quality was retained and the read with adapters was removed. (3) SOAPsnp V1.05 was used to assemble the consensus sequence and call genotypes. (4) The read with alignment length less than 75 bp was removed. The Unified Genotyper tool from GATK V1.0.4705 was applied to detect the small insertions and deletions (Indels) SNP and Indel detection were performed only on the targeted exome regions and flanking regions within 200 bp.

### 2.5. Filtering and Annotation

Three major steps were taken to prioritize all the high-quality variants among cataract-related genes: (i) variants within intergenic, intronic, and UTR regions and synonymous mutations were excluded from later analysis; (ii) variants with high frequency in dbSNP137 (http://www.ncbi.nlm.nih.gov/projects/SNP/), 1000 Genome project (ftp://ftp.1000genomes.ebi.ac.uk/vol1/ftp), YH Database (http://yh.genomics.org.cn/), HapMap Project (ftp://ftp.ncbi.nlm.nih.gov/hapmap), and our in-house database, which was generated by our laboratory using 1800 whole exome sequencing data, were further excluded.

### 2.6. Mutation Validation with Sanger Sequencing

The whole exome sequencing and data analysis has revealed a heterozygous mutation in* CRYBB2* gene. Then direct sequencing was utilized to identify the variation in the family members and the normal subjects. PCR primers aiming to amplify fragments flanking the candidate loci were synthesized by Invitrogen, Shanghai, China (*CRYBB2*-exon6-F: ctc gcctctctctctgtctg;* CRYBB2*-exon6-R: gacccacagcagacaagttg). The direct sequencing was conducted on the ABI 3730 Genetic Analyzer (Applied Biosystems) following the standard procedures and the data were analyzed via the Human Genomic Database.

## 3. Results

### 3.1. Clinical Date of the Family

**Figure 1:**
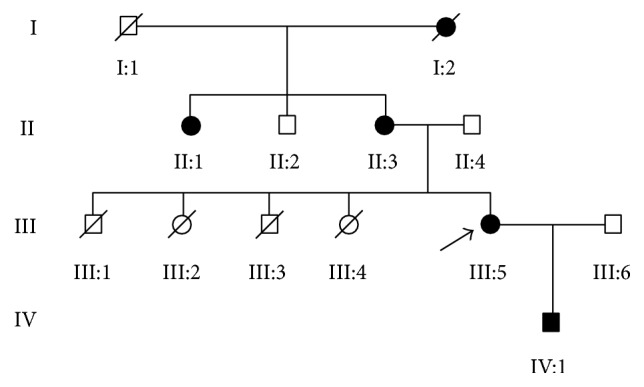
Pedigree of the Chinese family with congenital cataract. Squares and circles symbolize males and females, respectively. Clear and blackened symbols denote unaffected and affected individuals. The proband is marked with an arrow and the slash indicates deceased person. Three family members (III:5, III:6, and IV:1) participated in our study. The pedigree of the family suggests an autosomal dominant mode of inheritance.

**Figure 2:**
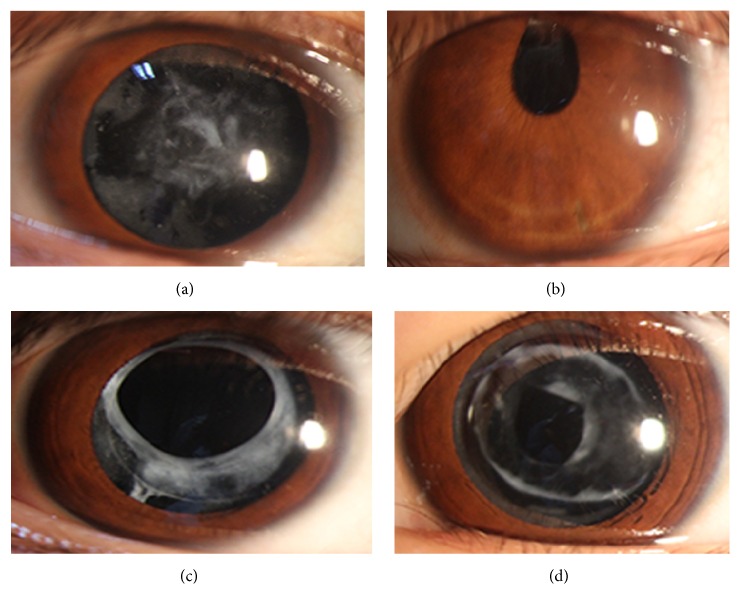
Slit-lamp photographs of the proband (III:5) and the proband's son (IV:1) in both eyes. The images (a) and (b) represent the left and right eyes of III:5, respectively. The images (c) and (d) correspond to left and right eyes of IV:1.

A four-generation family with congenital cataract from Sichuan Province of China including eight unaffected and five affected individuals were enrolled in our study. As the pedigree chart shown (Figure 1), the cataract exhibited an autosomal dominant inheritance pattern in the family. The proband (III:5), a 26-year-old female, was diagnosed with bilateral cataract in early childhood. Among the family members, the clinical information and blood samples of III:5 (the proband), III:6 (the husband of the proband, unaffected), and IV:1 (the son of the proband, affected) were available. The two affected members (the mother and her son) shared similar clinical features: an early-onset and significant loss of vision acuity (OD: 0.08/0.1, OS: 0.02/0.01, resp.), nystagmus, and strabismus in both eyes. The slit-lamp photographs of the two affected members in this family indicated that the lens were completely absorbed (Figure 2).

### 3.2. Whole Exome Sequencing and Data Analysis

The whole exome sequencing was performed at Axeq Technology Inc., Seoul, Korea, using the DNA sample from the proband (III:5). And we identified a total of 20726 SNPs in coding regions (9475 nonsynonymous SNPs, 10852 synonymous SNPs, and 399 other types of SNPs) and 495 coding Indels that may affect amino acid sequence using the mean read depth of target regions (62.3x) (Table 2). It is well known that functional SNP/Indel, mainly including nonsynonymous variants (NS), splice acceptor and donor site mutations (SS), and frameshift coding-region insertions or deletions (Indels), are more likely to be the disease-causing mutations. In our study, we have successfully identified 6315 functional SNPs and 231 functional Indels. We compared these variants with the known cataract genes and 41 variants were found. We then subsequently compared these variants in the proband with the dbSNP137, 1000 Genomes Project, HapMap project, YH database, and our in-house generated database using 1800 whole exome sequencing pieces of data, of which, the in-house data was chiefly applied to exclude the variants with high frequency in normal controls. Based on the autosomal dominant mode of inheritance, we finally shirked down the filtered data to one heterozygous variant, the heterozygous mutation c.499T<G (p.E167X) in* CRYBB2* gene.

### 3.3. Mutation Detection and Analysis

**Figure 3:**
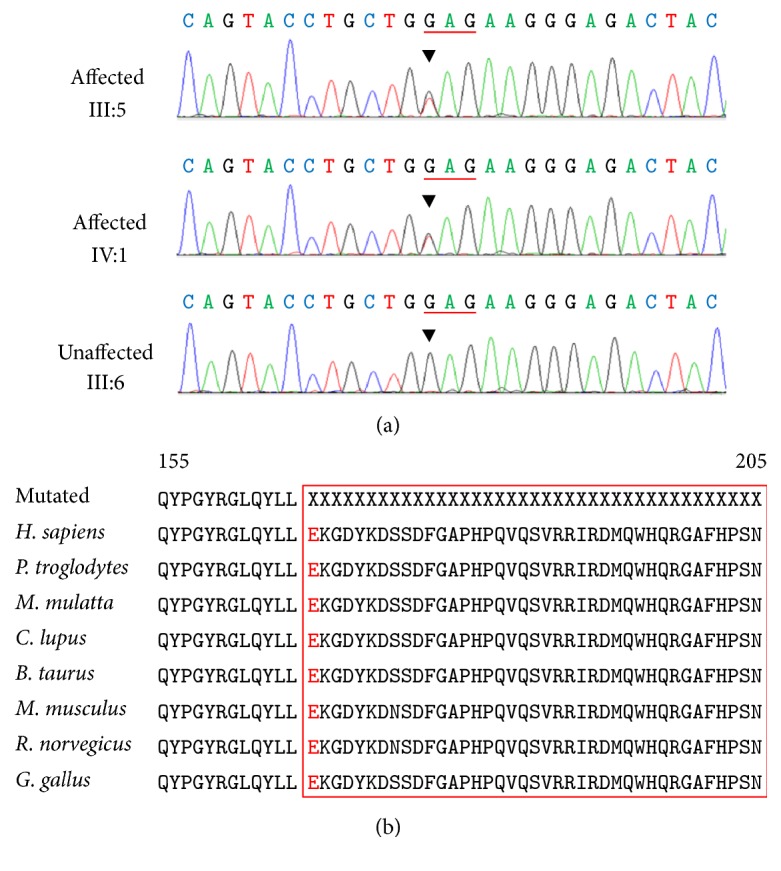
Representative chromatogram of* CRYBB2* sequence. (a) Sanger sequencing analysis of the affected and unaffected individuals in the ADCC Chinese family, showing a heterozygous mutation (c.499G>T) in exon 6 of* CRYBB2* (black triangles). Moreover, this transition resulted in a stopgain mutation. (b) Multiple-sequence alignment in* CRYBB2* from different species reveals that codon 167, where the mutation (p.E167X) occurred, is highly conserved (highlighted in red box).

Direct sequencing was used to further identify the variation of* CRYBB2* in the family members including III:5 (the proband), III:6 (the husband of the proband, unaffected), and IV:1 (the son of the proband, affected). The heterozygous mutation c.499T<G (p.E167X) was carried by the mother III:5 and her son IV:1, while her husband III:6 did not carry this mutation (shown in Figure 3(a)). Sanger sequencing analysis showing the heterozygous variant c.499T<G (p.E167X) cosegregated with the phenotype. Meanwhile, the mutation was not identified in the 1000 normal controls by Sanger sequencing. We also checked the mutation in the newly available ExAC database of 63,000 control exomes (http://exac.broadinstitute.org/) and no variants were reported in this locus of* CRYBB2 *gene. Taken together, these results including the clinical information indicated that the heterozygous mutation c.499T<G (p.E167X) in the exon 6 of* CRYBB2* gene is the causal mutation responsible for congenital cataract in this family.

According to GenBank accession number NM_000487.1, the novel stopgain mutation c.499T<G (p.E167X) in* CRYBB2* gene found in our study eventually introduced a loss of 39 amino acids from *β*B2-crystallin (including 205 amino acids). As shown in Figure 3(b), the amino acid changes affected highly conserved residues in eight species which might influence the formation of the fourth Greek key in *β*B2-crystallin. Therefore, the novel stopgain mutation was likely to affect the function of* CRYBB2* protein.

## 4. Discussion

Congenital cataract, characterized by clinical and genetical heterogeneity, is mainly autosomal dominant inheritance [6]. The genetic factor can account for about one-third of congenital cataract cases [10]. With the development of molecular genetic techniques in recent decades, accumulating groups focus on the field of the genetic defects about congenital cataract. In this study, we have identified a novel mutation c.499T<G (p.E167X) in the exon 6 of* CRYBB2* in a four-generation Chinese family affected with autosomal dominant congenital cataract by the whole exome sequencing and direct Sanger sequencing.

In the human lens, over 90% of lens soluble proteins are crystalline proteins including *α*-, *β*-, and *γ*-crystallins with a proportion of 40%, 35%, and 25%, respectively. The early studies have revealed that the stability, ratio, and spatial sequence of crystallin proteins played a critical role in the lens transparency and light transmission [40, 42–46]. Among them, *β*B2-crystallin encoded by* CRYBB2 *gene is the most abundant and most soluble *β*-crystallin in the lens [47]. As previously reported, scientists have already found that* CRYBB2* gene, belonging to the crystallin genes, can account for approximately half cataract families with known mutations [48]. And in recent years, increasing mutations in the* CRYBB2* gene, especially in the last two exons area, were found and reported to be associated with variable type of congenital cataract in different families and population.

The* CRYBB2 *gene (NM_000496) spanning 12.22 kb on chromosome 22q11.23 encodes *β*B2-crystallin protein containing 205 amino acids in human. It consists of six exons and is regarded as one of the most important genes for lens transparency. Among those exons, the first exon is not translated, the second encodes the NH2-terminal extension, and the remaining four exons contribute to one “Greek key” motif, respectively [49]. The *β*B2-crystallin protein is a member of the *β*-crystallin family and it is mainly expressed in human lens which can also be found in retina and brains [50]. The *β*B2-crystallin protein has four “Greek key” motifs in which the first two (1 and 2) and the last two (3 and 4) are, respectively, in the NH2- and COOH-terminal domain [51]. Meanwhile *β*B2-crystallin protein, as the major form of *β*-crystallin, is not only the most soluble but also the least changed crystallin protein in a lifetime [47, 52]. Therefore, the solubility and arrangement of *β*B2-crystallin play an important role in the development of human lens.

**Figure 4:**
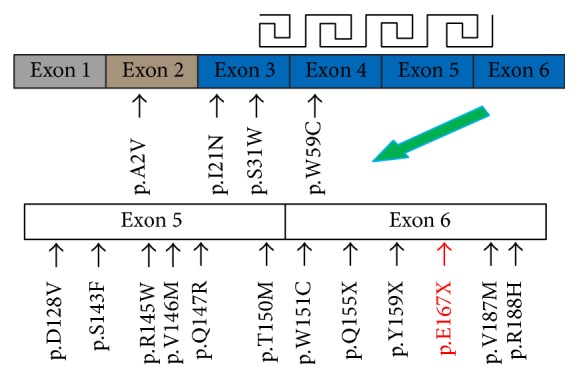
Schematic overview of gene structure and missense mutations found in* CRYBB2*. The* CRYBB2* gene consists of six exons; each of the exons (exon 3 to exon 6) encodes one Greek key motif. The mutation identified in this study is highlighted in red.

It is estimated that crystallin gene mutations can be responsible for approximately half of congenital cataract family [48]. And so far, at least fifteen mutations in the* CRYBB2* gene which belongs to a member of crystallin genes have been uncovered with respect to several phenotypes of congenital cataract in different families and population, such as W59C, Q155X, and V187M (Table 3) [6, 8, 9, 26–41]. And we can know that most of the mutations in the* CRYBB2 *gene centrally located in the last two exons (Figure 4). For example, p.Q155X mutation in the exon 6 resulted in a loss of 51 amino acids of *β*B2-crystallin which has been identified in different families and population. Yao et al. found that p.Q155X mutation can influence the formation of the fourth Greek key motif in the *β*B2-crystallin as well as probably changing the folding properties of *β*B2-crystallin [8]. Based on the mammalian two-hybrid system assay, spectroscopy (circular dichroism and fluorescence), and FPLC chromatography, scientists also discovered that p.Q155X mutation had an effect on the biophysical properties of *β*B2-crystallin (the decreased ordered structure and stability) which might be responsible for the cataract formation [53].

In addition, some mutant lines in mice have been reported which could affect the *β*B2-crystallin such as Philly [54], Aey2 [55], and O377 [56] characterized by dominant, progressive cataracts. For example, O377 mutation (intron 5:_57 A->T) resulted in 19 additional amino acids in front of the fourth Greek key motif which might form an additional loop near the carboxyl terminus for *β*B2-crystallin [56]. Meanwhile, the* Crybb2* knockout mice could develop cataracts 6–8 weeks after birth and cataract severity increased with age [57]. And we need further studies about* Crybb2 *animal models to elucidate the underlying molecular mechanism of which the* CRYBB2* mutations contribute to the cataract. The* CRYBB2* gene, in summary, plays a significant role both in the development of human and mice lens and it might be a critical region susceptible for mutations leading to lens opacity.

Likewise, the p.E167X mutation identified in our paper led to a truncation of 39 amino acids from the COOH-terminal end of *β*B2-crystallin. This mutation occurred in a highly conserved region of the exon 6 which would influence the length of the COOH-terminal arm and the molecular weight of *β*B2-crystallin protein. This alteration might affect the formation of the fourth Greek key in *β*B2-crystallin. The early studies have provided some evidence that *β*B2-crystallin played an important role in all three *β*-crystallin aggregates, especially the C- and N-terminal extension [58]. Based on these studies, we could make such an assumption that the mutation p.E167X identified in this study would not be conducive to anchoring *β*B2 subunit itself and the higher aggregation potentially resulting in a misfolded protein. In addition, some groups found that mutations may change the protein-protein interactions and further affect protein solubility and lens transparency [59]. All these studies provide us with new research directions. For the limitation of the animal model in the present study, the pathological mechanisms and functional study of* CRYBB2* p.E167X causing congenital cataract would be further identified.

In conclusion, a novel stopgain mutation (p.E167X) in the exon 6 of* CRYBB2* was identified in a four-generation Chinese family with ADCC and this mutation was likely to affect the formation of the fourth Greek key of *β*B2-crystallin. Our findings could not only provide some evidence for the importance of *β*B2-crystallin in the formation of cataract but also expand the mutation spectrum of* CRYBB2* gene in Chinese congenital cataract population. In the future, function studies are needed to evaluate the definite molecular mechanism resulting from the p.E167X mutation.

## Figures and Tables

**Table 1:** Clinical information about three members in the family.

ID	Age/gender	Relationship	Type of cataract	Onset age (months)	Surgery age (year)	Visual acuity		Mutation	Mutation type
						OD	OS		
III:5	26/F	Proband	ADCC	Since birth	9 (left eye)	0.08	0.02	c.499T<G p.E167X	Het
III:6	44/M	Proband' husband	Normal	Normal	Normal	1.0	1.0	—	—
IV:1	8/M	Proband' son	ADCC	Since birth	2 (both eyes)	0.1	0.01	c.499T<G p.E167X	Het

**Table 2:** Number of candidate SNPs/Indels filtered against several public variation databases and the in-house data.

	Feature_SNPs and Indels in patient III:5 of family ADCC
Total_SNPs^*∗*1^	69447
Total Indels^*∗*2^	6873
Coding_SNP/Indels^*∗*3^	20726/495
Functional_SNP/Indels^*∗*4^	6315/231
Filtered_known gene^*∗∗*^	41
Filtered_DBsnp137common/indel^#1^	
Filtered_DBsnp/indel_1000gene(2011)^#2^	38
Filtered_DBsnp_1000gene_Hapmap_YH^#3^	
Filtered in House Data^##^	4
heterozygous	1

Total_SNPs^*∗*1^, Total Indels^*∗*2^, Coding_SNP/Indels^*∗*3^, and Functional_SNP/Indels^*∗*4^: the date was provided by the “human exome capture sequencing date analysis report” and we made a statistical analysis about them.

Filtered_known gene^*∗∗*^: we filtered known gene mainly based on the related references.

Filtered_DBsnp137common/indel^#1^, Filtered_DBsnp/indel_1000gene(2011)^#2^, and Filtered_DBsnp_1000gene_Hapmap_YH^#3^: they are some public databases and all the versions of databases used in our paper were the latest versions. The access date in which we accessed the public databases was about in April 2016.

Filtered in House Data^##^: it is our lab's database which was generated by our laboratory using 1800 whole exome sequencing data.

**Table 3:** Mutations previously described in the *CRYBB2 *gene associated with congenital cataracts in human.

Bp exchange	Aa exchange	Biologic consequence	Origin of family	Reference
c.C5T	p.A2V	Posterior subcapsular	Chinese	[26]
c.T62A	p.I21N	ADCC	Chinese	[27]
c.C92G	p.S31W	Coronary cataract	Chinese	[28]
c.G54A	p.(=)	ADCC	Indian	[29]
	p.W59C	ADCC	Indian	[29]
c.A383T	p.D128V	ADCC	German	[30]
c.C428T	p.S143F	ADCC	Italian	[31]
c.C433T	p.E145W	ADCC	Danish/German	[32, 33]
c.G436T	p.V146M	ADCC	Chinese	[27]
c.A440G	p.Q147R	ADCC	Danish/German	[32, 33]
c.C449T	p.T150M	ADCC	Danish/German	[32, 33]
c.G465T	p.W151C	ADCC	Chinese	[34]
		Central nuclear	Indian	[35]
c.C475T	p.Q155X	Sutural opacity and fish tail-like branches	American	[36]
		Cerulean	American	[37]
		ADCC	Canadian	[6]
		ADCC	Chilean	[38]
		Progressive polymorphic coronary ADCC	Indian	[9]
		Cerulean ADCC	Chinese	[39]
		Progressive polymorphic	Chinese	[8]
c.C477A	p.Y159X	ADCC	Danish	[32]
c.G607A	p.V187M	Nuclear cataract	Basotho	[40]
c.G563A	p.R188H	ADCC	German	[41]
